# Development and evaluation of a standardized ELISA for the determination of autoantibodies against cN-1A (Mup44, NT5C1A) in sporadic inclusion body myositis

**DOI:** 10.1007/s13317-016-0088-8

**Published:** 2016-11-17

**Authors:** Sabine L. Kramp, Dmitry Karayev, Guo Shen, Allan L. Metzger, Robert I. Morris, Eugene Karayev, Yvonne Lam, Richard M. Kazdan, Ger J. M. Pruijn, Sandra Saschenbrecker, Cornelia Dähnrich, Wolfgang Schlumberger

**Affiliations:** 1 Institute for Experimental Immunology, Euroimmun AG, Seekamp 31, 23560 Lübeck, Germany; 2 RDL Reference Laboratory Inc., 10755 Venice Blvd., Los Angeles, CA 90034 USA; 3 Department of Biomolecular Chemistry, Institute for Molecules and Materials and Radboud Institute for Molecular Life Sciences, Radboud University Nijmegen, P.O. Box 9101, 6500 HB Nijmegen, The Netherlands

**Keywords:** Anti-cN-1A, Anti-Mup44, Autoantibodies, Autoimmunity, ELISA, Myopathy, Sporadic inclusion body myositis

## Abstract

**Purpose:**

Sporadic inclusion body myositis (sIBM) is an autoimmune degenerative disease of the muscle, with inflammatory infiltrates and inclusion vacuoles. Its pathogenesis is not fully understood and the diagnosis is hampered by its imprecise characteristics, at times indistinguishable from other idiopathic inflammatory myopathies such as polymyositis and dermatomyositis. The diagnosis may be assisted by the detection of autoantibodies targeting Mup44, a skeletal muscle antigen identified as cytosolic 5′-nucleotidase 1A (cN-1A, NT5C1A). A novel standardized anti-cN-1A IgG ELISA was developed and its diagnostic performance was evaluated by two reference laboratories.

**Methods:**

Recombinant human full-length cN-1A was expressed and purified, and subsequently utilized to set up a standardized ELISA. To evaluate the novel assay, laboratory A examined sera from North American patients with clinically and pathologically diagnosed definite sIBM (*n* = 17), suspected sIBM (*n* = 14), myositis controls (*n* = 110), non-myositis autoimmune controls (*n* = 93) and healthy subjects (*n* = 52). Laboratory B analyzed a Dutch cohort of definite sIBM patients (*n* = 51) and healthy controls (*n* = 202).

**Results:**

Anti-cN-1A reactivity was most frequent in definite sIBM (39.2–47.1%), but absent in biopsy-proven classic polymyositis or dermatomyositis. Overall diagnostic sensitivity and specificity amounted to 35.5 and 96.1% (laboratory A) and 39.2 and 96.5% (laboratory B).

**Conclusions:**

Anti-cN-1A autoantibodies were detected by ELISA with moderate sensitivity, but high specificity for sIBM and may therefore help diagnose this infrequent and difficult-to-diagnose myopathy. The novel anti-cN-1A IgG ELISA can improve and accelerate the diagnosis of sIBM using sera where muscle biopsy is delayed or unfeasible.

## Introduction

Sporadic inclusion body myositis (sIBM) is the most common acquired myopathy in patients over 50 years old [1, 2]. sIBM has a male predominance and a prevalence of 1–71 people per million individuals, rising up to 139 per million among people aged above 50 years and varying between different populations [3–10]. Its primary cause is still controversial, but autoimmune and degenerative processes are assumed to be pathogenically relevant, involving a complex interaction between, for example, environmental triggers, genetic susceptibility, aging, oxidative and endoplasmic reticulum stress, mitochondrial abnormalities and aberrant myoproteostasis [11–13].

sIBM is classified as an idiopathic inflammatory myopathy (IIM), along with polymyositis, dermatomyositis and immune-mediated necrotizing myopathies [14]. The clinical manifestations of sIBM are typically related to muscle weakness and atrophy, preferentially affecting the quadriceps femoris, as well as the wrist and finger flexors. Other muscle groups (e.g., finger extensors, upper arm muscles, ankle dorsiflexors, paraspinal, facial and oropharyngeal muscles) can also be affected to varying degrees. Asymmetrical involvement is common, with the non-dominant side showing more severe manifestations in most cases. The presenting symptoms include, among others, motor deficits (e.g., difficulties in standing up, climbing stairs, walking, gripping), falls, foot drop, muscle wasting and dysphagia. The disease course is chronic and slowly progressive, leading to severe disability, often with wheelchair dependency [15–17]. Muscle tissue in sIBM is characterized by inflammatory and degenerative changes, such as endomysial inflammatory infiltrates (macrophages, CD8+ T cells), myofiber MHC class I and II upregulation, rimmed vacuoles, congophilic inclusions, sarcoplasmic protein aggregates (e.g., amyloid-β, p62, TDP-43) and mitochondrial abnormalities (e.g., cytochrome c oxidase negative myofibers) [15, 18, 19]. However, due to their patchy distribution and absence in early disease stages, some of these histological markers are not detectable in up to 30% of patients with clinically typical sIBM, thus causing diagnostic difficulty and delay [2, 20–22].

The diagnosis of sIBM is suspected on clinical grounds and confirmed by the above-mentioned key pathological features on muscle biopsy [23–27]. In addition, magnetic resonance imaging, electromyography and laboratory testing (e.g., serum creatine kinase level, autoantibodies) can aid the diagnosis [15, 28].

In 2013, the target of the sIBM-associated anti-Mup44 autoantibodies was identified as cytosolic 5′-nucleotidase 1A (cN-1A, cN1A, NT5C1A, Mup44) [29–31], a 41 kDa enzyme that is most abundant in the skeletal muscle, catalyzing nucleotide hydrolysis and regulating nucleotide metabolism [32, 33]. In patients with sIBM, the aberrant accumulation of cN-1A in areas of myofiber degeneration may contribute to its antigenicity, providing a plausible link between myodegenerative and autoimmune features in disease pathogenesis [30]. Anti-cN-1A autoantibodies are currently the only known serum biomarker for sIBM [2] and have the potential of early diagnosis and improving the management of patients with suspected sIBM. This is particularly important considering an initial misdiagnosis rate of approximately 30–53%, a mean delay to diagnosis of 4.9–8 years [7, 17, 34] and, in many cases, the need for multiple muscle biopsies before it is possible to establish the correct diagnosis [20, 21, 35]. In particular, the clinical distinction between sIBM and polymyositis can be challenging, the latter being the most common initial misdiagnosis. Recent studies have reported anti-cN-1A reactivity in the serum of 33–76% patients with sIBM, but in not more than 5% of patients with polymyositis, with an overall specificity varying between 87 and 100% depending on the methodology and cohorts studied [29–31, 36–39]. Thus, the determination of anti-cN-1A autoantibodies is useful for distinguishing sIBM from other inflammatory myopathies. Such a distinction is crucial for therapeutic decisions since sIBM, in contrast to polymyositis, is poorly responsive to conventional immunotherapies [40].

In this study, the diagnostic performance of a novel, commercially available ELISA for the standardized detection of anti-cN-1A autoantibodies was evaluated at two reference laboratories using serum panels from clinically and pathologically characterized sIBM patients and various controls. Additionally, the assay was compared to established in-house tests.

## Methods

### Patients and serum samples

Serum samples collected at the Rheumatology Diagnostics Laboratory (RDL Inc., Los Angeles, CA, USA) were obtained from North American patients with clinically and pathologically diagnosed definite sIBM (*n* = 17), suspected sIBM (*n* = 14) and other myopathies (*n* = 110) [including polymyositis (*n* = 7), dermatomyositis (*n* = 4), unspecified myositis without sIBM (*n* = 94), muscle atrophy (*n* = 1) and myonecrosis (*n* = 4)]. The diagnosis of sIBM was established according to the European Neuromuscular Centre (ENMC) criteria [26]. The Bohan and Peter’s criteria were used for the classification of polymyositis and dermatomyositis [41]. Sera from patients clinically and serologically diagnosed with systemic lupus erythematosus (*n* = 33), scleroderma (*n* = 20), Sjögren’s syndrome (*n* = 20) and rheumatoid arthritis (*n* = 20) as well as healthy subjects (*n* = 52) were also collected at the same center and included as controls.

Sera from two other cohorts were analyzed in parallel, including samples from Dutch patients with clinically and pathologically diagnosed definite sIBM (*n* = 51) obtained from the Radboud University Medical Center (Nijmegen, The Netherlands) and samples from healthy controls (*n* = 202) collected at the University Medical Center Schleswig–Holstein, Campus Lübeck (Lübeck, Germany). The Dutch sIBM patients fulfilled the ENMC criteria for sIBM [27], while poly- and dermatomyositis were differentiated based on the diagnostic criteria of Tanimoto et al. [42].

Serum samples were tested anonymously to maintain confidentiality. All study procedures were in accordance with the ethical standards of the institutional research committees and with the Helsinki Declaration.

### Heterologous expression of recombinant human cN-1A

cDNA encoding full-length human cN-1A according to UniProt accession number Q9BXI3 was integrated in the prokaryotic expression vector pET24d (Novagen, Darmstadt, Germany). The protein with a C-terminal hexa-His tag was produced in *Escherichia coli* RosettaBlue(DE3)pLacI (Novagen) and purified using Ni-NTA affinity chromatography (Qiagen, Hilden, Germany) as described [43]. The purified antigen was analyzed by denaturing electrophoresis using the NuPAGE Novex 4–12% Bis–Tris gradient gel system according to the manufacturer’s instructions (Invitrogen, Karlsruhe, Germany) and by MALDI-TOF mass spectrometry [44].

### Anti-cN-1A IgG ELISA

Microtiter plates (Nunc, Roskilde, Denmark) were coated with cN-1A (2.5 µg/ml in PBS, pH 7.5) overnight at 4 °C, washed with PBS containing 0.05% (v/v) Tween-20 and blocked with 0.1% (w/v) casein in PBS for 2 h, followed by another washing step. The determination of anti-cN-1A autoantibodies was performed according to the manufacturer’s instructions (Euroimmun, Lübeck, Germany) using the coated microtiter plates and standardized reagents provided with the test kits. Briefly, sera diluted 1:101 in the sample buffer were applied to the wells and incubated for 30 min. Bound antibodies were detected by adding rabbit anti-human IgG peroxidase conjugate for 30 min, followed by staining with tetramethylbenzidine for 15 min. The enzymatic reaction was stopped with 0.5 mol/l sulfuric acid. The optical density (OD) was measured photometrically at 450 nm (reference 620 nm). A calibrator was included in each run. As a result of receiver-operating characteristic (ROC) analysis for the assessment of assay accuracy, OD450 values of patient samples equal to or greater than the OD450 value of the calibrator (cutoff) were considered positive [ratio (OD450 sample/OD450 calibrator) ≥1.0]. At RDL, an in-house calibrator was utilized for the ratio calculation (OD450 sample/OD450 calibrator). Then a unit multiplier was used to determine the arbitrary units. The unit cutoff was based on the mean of 52 healthy individuals plus three standard deviations (mean + 3 SD). The results of less than 25 units were considered negative.

### Peptide ELISA

ELISAs based on three synthetic 23-mer peptides corresponding to the major epitope regions of cN-1A were performed as described previously [38]. Sera were assessed as reactive if their OD450 value was above the established cutoff value for at least one of the peptide antigens (peptide 1, OD450 ≥ 0.384; peptide 2, OD450 ≥ 0.270; peptide 3, OD450 ≥ 0.280). For each serum sample, only the maximum OD450 value of the three peptide ELISAs (maximum peptide reactivity) was used for correlation analysis.

### Immunoprecipitation assay

Immunoprecipitation was performed with radiolabeled in vitro translated full-length cN-1A, followed by SDS-PAGE and quantification by phosphorimaging, as described previously [31]. To differentiate between high and low cN-1A immunoprecipitation efficiencies, cutoff levels of 5 and 1% (fraction of input cN-1A precipitated) were arbitrarily chosen.

### Determination of other autoantibodies

Autoantibodies against myositis-related autoantibodies were determined using the Euroline Autoimmune Inflammatory Myopathies 16 Ag (IgG) immunoblot according to the instructions of the manufacturer (Euroimmun, Lübeck, Germany), allowing monospecific detection of Ro-52, isoleucyl-tRNA synthetase (OJ), glycyl-tRNA synthetase (EJ), alanyl-tRNA synthetase (PL-12), threonyl-tRNA synthetase (PL-7), 54 kDa recombinant signal recognition particle (SRP), histidyl-tRNA synthetase (Jo-1), PM-Scl75, PM-Scl100, Ku, SUMO activating enzyme subunits 1 (SAE1), MJ-p140-MU 140 kD protein/MORC3 (NXP2), melanoma differentiation-associated gene 5 (MDA5), transcriptional intermediary factor 1-gamma (TIF1γ), chromodomain-helicase-DNA-binding protein 4 (Mi-2β) and chromodomain-helicase-DNA-binding protein 3 (Mi-2α).

### Statistical analysis

Statistical analyses were performed using GraphPad Prism 6 (GraphPad Software Inc., La Jolla, CA, USA) and SigmaPlot 13.0 analysis software (SSI, San Jose, CA, USA). Confidence intervals (CI 95%) were calculated according to the modified Wald method. The degree of correlation was evaluated by calculating the Pearson’s or the Spearman’s rank order correlation coefficient (*r*). *P* values <0.05 were considered to be significant.

## Results

**Fig. 1:**
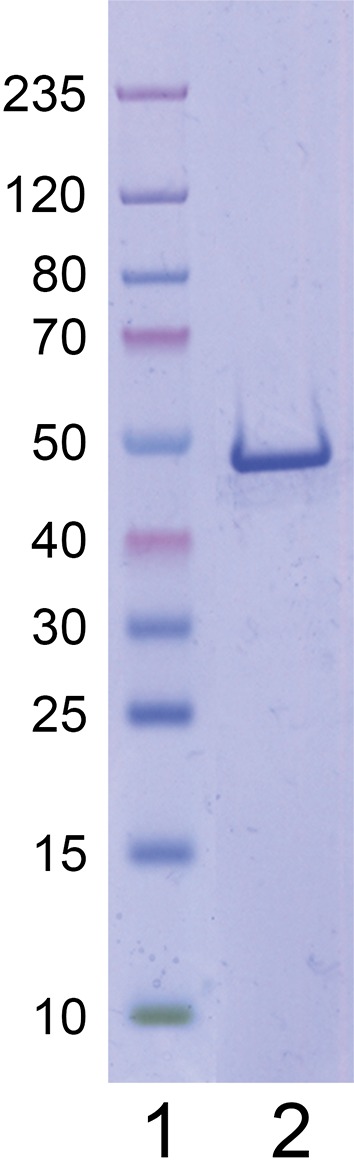
Analysis of purified recombinant cN-1A. Molecular weight markers (*lane 1*) and 1 µg of purified cN-1A (*lane 2*) were separated by sodium dodecyl sulfate–polyacrylamide gel electrophoresis followed by Coomassie staining. The molecular masses (kDa) of the size markers are indicated on the *left*

Recombinant full-length human cN-1A was expressed in *E. coli* as His-tagged protein and purified by immobilized metal chelate affinity chromatography. When separated by SDS-PAGE, the protein migrated consistent with its calculated molecular mass of 41 kDa (taking into account the additional mass resulting from fused amino acids encoded by the expression vector, such as the His tag) and showed a high degree of purity (Fig. 1). MALDI-TOF mass spectrometry confirmed protein identity. A semi-quantitative ELISA based on the purified antigen was developed for the standardized detection of serum anti-cN-1A IgG autoantibodies. The diagnostic performance of this assay was studied at two independent reference laboratories.

At the RDL Reference Laboratory (Los Angeles, CA, USA), reactivity against cN-1A was detected in 8 (47.1%) of 17 North American patients with definite sIBM and in 3 (21.4%) of 14 patients with suspected sIBM. Among 110 patients with non-sIBM myopathies (myositis controls), positive reactivity was found in 4 (3.6%) cases, all of which had been classified as having unspecified myositis without sIBM. In contrast, all patients with biopsy-proven classical polymyositis, dermatomyositis, muscle atrophy or myonecrosis were found to be anti-cN-1A negative. Out of 93 sera from other autoimmune disease controls, 5 (5.4%) showed positive reactivity, of which 2 were from patients with systemic lupus erythematosus, 2 with scleroderma and 1 with rheumatoid arthritis. None of the patients with Sjögren’s syndrome and only 1 (1.9%) of 52 healthy controls were found to be positive. Referring to the total of 31 patients with definite or suspected sIBM and 255 controls, the diagnostic sensitivity and specificity of this novel ELISA amounted to 35.5 and 96.1%, respectively (Table 1).

**Table 1:** Anti-cN-1A reactivity in sera from patients with sporadic inclusion body myositis, disease controls and healthy controls as determined by anti-cN-1A IgG ELISA at the RDL Reference Laboratory (Los Angeles, CA, USA)

Cohorts	Subgroups	*n*	Anti-cN-1A ELISA (IgG)			
			Positive	Negative	Sensitivity (CI 95%)	Specificity (CI 95%)
sIBM	Definite sIBM	17	8	9	47.1% (26.2–69.0%)	
	Suspected sIBM^a^	14	3	11	21.4% (6.8–48.3%)	
Total sIBM		31	11	20	35.5% (21.1–53.1%)	
Myositis controls	Polymyositis	7	0	7		100.0% (59.6–100.0%)
	Dermatomyositis	4	0	4		100.0% (45.4–100.0%)
	Unspecified myositis without sIBM^b^	94	4	90		95.7% (89.2–98.7%)
	Muscle atrophy	1	0	1		100.0% (16.8–100.0%)
	Myonecrosis	4	0	4		100.0% (45.4–100.0%)
Other autoimmune disease controls	Systemic lupus erythematosus	33	2	31		93.9% (79.4–99.3%)
	Scleroderma	20	2	18		90.0% (68.7–98.4%)
	Sjögren’s syndrome	20	0	20		100.0% (81.0–100.0%)
	Rheumatoid arthritis	20	1	19		95.0% (74.6–100.0%)
Healthy controls		52	1	51		98.1% (88.9–100.0%)
Total controls		255	10	245		96.1% (92.8–98.0%)

*CI* confidence interval, *cN-1A* cytosolic 5′-nucleotidase 1A, *sIBM* sporadic inclusion body myositis

^a^Suspected sIBM include biopsy readings of possible, probable and doubtful sIBM patients

^b^Unspecified myositis without sIBM include idiopathic inflammatory myopathy (polymyositis, dermatomyositis and uncharacterized myositis) with no available biopsy data for this study

In parallel, two further cohorts were analyzed by means of the anti-cN-1A IgG ELISA at the Institute for Experimental Immunology (Lübeck, Germany). Here, reactivity against cN-1A was present in 20 (39.2%) of 51 Dutch patients with definite sIBM and in 7 (3.5%) of 202 healthy subjects, resulting in a sensitivity of 39.2% and specificity of 96.5% (Table 2).

**Table 2:** Anti-cN-1A reactivity in sera from patients with sporadic inclusion body myositis (sIBM) and in healthy controls as determined by anti-cN-1A IgG ELISA at the Institute for Experimental Immunology (Lübeck, Germany)

Cohorts	*n*	Anti-cN-1A ELISA (IgG)			
		Positive	Negative	Sensitivity (CI 95%)	Specificity (CI 95%)
sIBM	51	20	31	39.2% (27.0–52.9%)	
Healthy controls	202	7	195		96.5% (92.9–98.5%)

*CI* confidence interval, *cN-1A* cytosolic 5′-nucleotidase 1A, *sIBM* sporadic inclusion body myositis

**Fig. 2:**
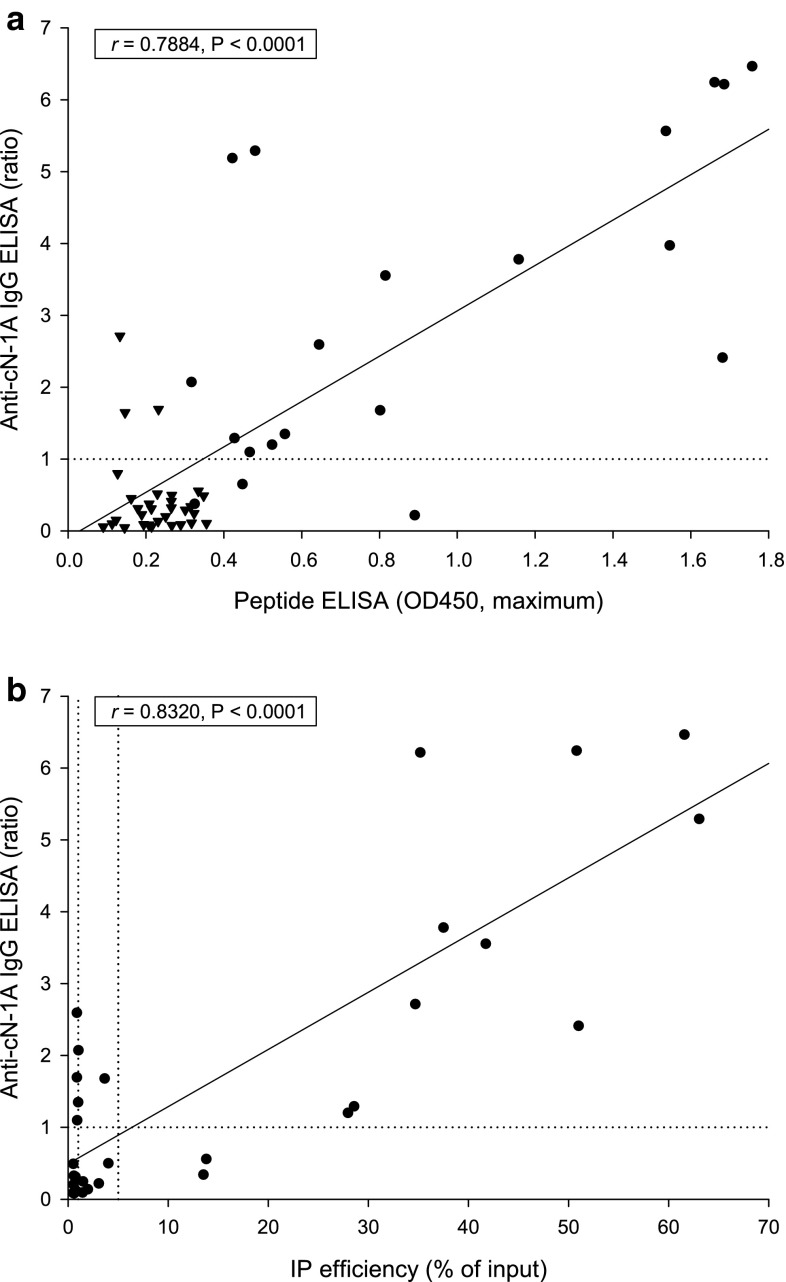
Correlation analysis. Reactivity of serum samples from Dutch patients with sporadic inclusion body myositis was measured by anti-cN-1A IgG ELISA (Euroimmun) based on recombinant full-length cN-1A, by ELISAs based on three synthetic peptides representing major cN-1A epitopes (*n* = 51), and by immunoprecipitation (IP) of in vitro translated full-length cN-1A (*n* = 30). Results by anti-cN-1A IgG ELISA were plotted against **a** the maximum OD450 values measured by either of the three peptide ELISAs and **b** against the efficiency of immunoprecipitation. *Dotted lines* represent cutoff values (anti-cN-1A IgG ELISA, ratio ≥1.0; IP high efficiency, >5% of input; IP low efficiency, >1% of input). In panel a, *triangles*/*circles* depict serum specimens with a maximum OD450 value below/above the cutoff value of the respective peptide ELISA (peptide 1, OD450 ≥ 0.384; peptide 2, OD450 ≥ 0.270; peptide 3, OD450 ≥ 0.280). Correlation was evaluated by Pearson’s correlation coefficients (*r*) as indicated. *P* values <0.05 were considered significant

The Dutch patients’ sera were also examined for anti-cN-1A reactivity using previously published ELISAs based on three synthetic peptides representing immunodominant cN-1A epitopes, and for their efficiency to immunoprecipitate recombinant in vitro translated full-length cN-1A. As depicted in the scatter plots in Fig. 2, there was a significant correlation of the novel anti-cN-1A IgG ELISA with reactivity measured by the peptide ELISA (*r* = 0.7884, *P* < 0.0001) and the immunoprecipitation assay (*r* = 0.8320, *P* < 0.0001).

Testing for myositis-related autoantibodies in a subset of 75 sIBM sera revealed reactivity against Ro-52 in 14.7%, against PL-7, PL-12, SRP and Ku in 4% each, against TIF1γ in 2.7%, and against OJ, EJ, Jo-1, PM-Scl75, PM-Scl100, and Mi-2β in 1.3% each (Table 3).

**Table 3:** Reactivity of myositis-related autoantibodies in sera from patients with sporadic inclusion body myositis (sIBM) as determined by Euroline Autoimmune Inflammatory Myopathies 16 Ag (IgG) immunoblot (Euroimmun)

Myositis-related autoantibodies	sIBM patients				
	All (*n* = 75)		Anti-cN-1A-positive (*n* = 28)		Correlation to anti-cN-1A
	Positive		Positive		
	*n*	Frequency	*n*	Frequency	*r*
Anti-Ro-52	11	14.7%	9	32.1%	0.381^a^
Anti-OJ	1	1.3%	1	3.6%	0.312^c^
Anti-EJ	1	1.3%	0	0.0%	
Anti-PL-12	3	4.0%	2	7.1%	
Anti-PL-7	3	4.0%	1	3.6%	
Anti-SRP	3	4.0%	3	10.7%	0.315^b^
Anti-Jo-1	1	1.3%	0	0.0%	
Anti-PM-Scl75	1	1.3%	0	0.0%	
Anti-PM-Scl100	1	1.3%	1	3.6%	
Anti-Ku	3	4.0%	2	7.1%	
Anti-SAE1	0	0.0%	0	0.0%	
Anti-NXP2	0	0.0%	0	0.0%	
Anti-MDA5	0	0.0%	0	0.0%	
Anti-TIF1γ	2	2.7%	1	3.6%	
Anti-Mi-2β	1	1.3%	0	0.0%	
Anti-Mi-2α	0	0.0%	0	0.0%	

*cN-1A* cytosolic 5′-nucleotidase 1A, *sIBM* sporadic inclusion body myositis

^a^ *P* < 0.001, Spearman’s rank order correlation

^b^ *P* < 0.01, Spearman’s rank order correlation

^c^ *P* < 0.01, Pearson’s correlation

Spearman’s rank order correlation revealed a moderate relationship between anti-cN-1A reactivity and the presence of Ro-52 (*r* = 0.381, *P* < 0.01) and SRP (*r* = 0.315, *P* < 0.001) autoantibodies in sIBM, whereas Pearson’s correlation revealed a moderate relationship between anti-cN-1A and OJ autoantibodies (*r* = 0.312, *P* < 0.01). No correlation was detected between anti-cN-1A and any other analyzed autoantibodies.

## Discussion

In 2013, cN-1A was identified as a major immune target in sIBM [30, 31]. Circulating autoantibodies against this muscle antigen represent a new serologic marker for diagnosing sIBM, which is at times clinically indistinguishable from other forms of IIM, such as polymyositis [2]. In the present study, anti-cN-1A IgG autoantibodies were detected by a newly developed commercial ELISA with a diagnostic sensitivity of 35.5–39.2%, reaching the highest levels (47.1%) in a subgroup of patients with definite sIBM and a specificity of more than 96%. Importantly, sensitivity and specificity measured at two different laboratories using different serum panels were highly similar. Our data compare well with previous reports where, depending on the methods and cohorts studied, anti-cN-1A reactivity had been detected in the serum of 33–76% of sIBM patients, with an overall specificity of 87–100% [29–31, 36–39] (Table 4). Noteworthy, assays with a high sensitivity (>50%) were either based on immunoblot analysis or on ELISA for combined Ig isotype detection (IgM, IgA and IgG) and, in most cases, were associated with lower levels of specificity (<95%) [36, 39].

**Table 4:** Diagnostic performance of anti-cN-1A assays for the diagnosis of sporadic inclusion body myositis

References	Method	Number of sIBM patients	Number of controls	Sensitivity	Specificity (non-sIBM muscle diseases)	Specificity (total controls)
Salajegheh et al. [29]	Western blot against human skeletal muscle extract	25	40	52%	100%	100%
Larman et al. [30]	Dot blot against a synthetic 36-amino acid cN-1A peptide (high cutoff)	47	153	34%^a^ (70%)^b^	98%^a^ (92%)^b^	99%^a^
Pluk et al. [31]	Immunoprecipitation with in vitro translated recombinant cN-1A (high cutoff)	94	172	33%^a^	96%^a^	97%^a^
Greenberg et al. [36]	ELISA (IgG) using recombinant cN-1A	50	155	51%^b^	ND	94%^b^
	ELISA (combined IgG/IgA/IgM) using recombinant cN-1A	50	155	76%^b^	ND	91%^b^
Goyal et al. [37]	Western blot against recombinant cN-1A expressed in HEK293 cells, ELISA using recombinant cN-1A	25	ND	72%	ND	ND
Herbert et al. [38]	ELISA using three synthetic cN-1A peptides (à 23 amino acids) covering the major immunodominant epitopes	238	524	37%	96%	94%
Lloyd et al. [39]	Western blot against recombinant cN-1A expressed in HEK293 cells	117	383	61%	87%	87%
Kramp et al. (this study)	ELISA (Euroimmun, IgG) using recombinant full-length cN-1A; Laboratory A	31	255	36%	96%	95%
	ELISA (Euroimmun, IgG) using recombinant full-length cN-1A; Laboratory B	51	202	39%	ND	97%

*cN-1A* cytosolic 5′-nucleotidase 1A, *HEK293* human embryonic kidney 293 cell line, *ND* not determined, *sIBM* sporadic inclusion body myositis

^a^Data based on a high cutoff point

^b^Data obtained by receiver-operating characteristics (ROC) curve analysis using a cutoff for optimal assay accuracy

The newly developed ELISA is based on full-length cN-1A antigen and represents the first fully standardized commercial assay for routine anti-cN-1A testing. Considering that the complete spectrum of linear and/or conformational immunodominant epitopes is yet to be identified, the use of the full-length antigen may be diagnostically advantageous compared to assays based on peptides. When the reactivity of the same patient samples in the novel ELISA was compared with that in previously published synthetic cN-1A peptide ELISAs and with their efficiency in immunoprecipitation of recombinant in vitro translated full-length cN-1A [31, 38], good correlations were observed. Besides its commercial availability, the advantages of the new assay are that, one, the full-length antigen is used, allowing the detection of autoantibodies to most, if not all, epitopes; two, only a single assay has to be performed to detect the antibodies, whereas the reactivity with three distinct antigenic peptides has to be determined in three separate ELISAs; three, no laborious and special infrastructure requiring procedures, such as those for immunoprecipitation, are required; and four, a standardized assay can be applied in different laboratories.

The moderate prevalence of anti-cN-1A autoantibodies in sIBM does not reduce their significance as a serologic marker. Similar or lower prevalence levels are also characteristic for other serologic parameters. For example, the prevalence of myositis-specific autoantibodies (MSA: anti-Jo-1, anti-Mi-2, anti-SRP, anti-PL-7, anti-PL-12, anti-EJ, anti-OJ) and myositis-associated autoantibodies (MAA: anti-SSA/Ro-52, anti-Ku, anti-PM/Scl75, anti-PM/Scl100) in polymyositis/dermatomyositis does not exceed a total of 34.4% (MSA) and 41.4% (MAA), respectively [45].

Reactivity to individual MSA and MAA with low frequencies in sIBM patients is in accordance with previous publications [46]. Detected correlations between reactivity to anti-cN-1A and other autoantibodies have to be considered with caution due to the small sample numbers, whereby the correlation between anti-cN-1A and anti-Ro-52 seems to be likely. Interestingly, a concurrence of anti-Ro-52 with anti-Jo-1 in idiopathic inflammatory myopathy was reported earlier, and anti-Ro-52 and anti-cN-1A might be coupled in a similar way [47].

There is not yet clear evidence regarding clinical or pathophysiological differences between anti-cN-1A-positive and -negative sIBM patients. So far, only one study has provided data indicating that anti-cN-1A seropositivity may be associated with a more aggressive form of IBM, including more prominent motor, bulbar and respiratory involvement [37].

In patients with biopsy-proven classic polymyositis or dermatomyositis, we observed a specificity of 100%, confirming the utility of anti-cN-1A autoantibodies in distinguishing sIBM from polymyositis (but given the small number of patients, these results have to be interpreted with caution). In a control group of 94 patients classified as having unspecified myositis without sIBM (no biopsy data available), anti-cN-1A reactivity was positive in 4 (4.3%) cases that were polymyositis or dermatomyositis. However, this is far below the prevalence observed in sIBM and consistent with recent studies where anti-cN-1A positivity was observed in not more than 5% of patients with polymyositis [30, 31, 38, 39].

Interestingly, in previous studies, anti-cN-1A autoantibodies were also found at a moderate prevalence in patients with Sjögren’s syndrome (23–36%) and systemic lupus erythematosus (14–20%) [38, 39]. This finding was not confirmed by the present study where anti-cN-1A reactivity was detectable in none of the patients with Sjögren’s syndrome and in not more than 6.1% of patients with systemic lupus erythematosus, although the relatively small patient numbers allow only limited conclusions. In addition, 5.0% of patients with rheumatoid arthritis and 10.0% of patients with scleroderma were found to be anti-cN-1A positive. This is, however, of minor relevance for clinical practice because differentiating patients with sIBM from those with Sjögren’s syndrome, systemic lupus erythematosus or other non-myositis autoimmune conditions is usually clinically and immunologically straightforward [38, 39].

Currently, the diagnosis of sIBM is mainly based on clinical features and histopathological analyses of muscle biopsy specimens [26]. Misdiagnosis and delayed diagnosis occur frequently [7, 17, 34]. The detection of anti-cN-1A autoantibodies may facilitate the early diagnosis of sIBM, especially when the clinical presentation is not distinctive and/or when typical pathological features are not yet detectable. Therefore, and considering that treatment options and responses differ between the different forms of myositis, serologic testing is highly beneficial to improve the care and management of patients with suspected sIBM.

In conclusion, a sensitive and highly specific ELISA for the standardized detection of anti-cN-1A autoantibodies in sIBM has been developed. This assay can support the differential diagnosis of sIBM, particularly the distinction from other inflammatory myopathies, using sera where muscle biopsy is delayed or unfeasible, thus improving patient care. Further studies are in progress to compare larger cohorts by standardized testing to fully investigate the prevalence of anti-cN-1A autoantibodies in myositis and other autoimmune diseases. In addition, studies addressing the association of anti-cN-1A autoantibodies with clinical features in large patient groups are currently being performed and will provide more insight into the clinical meaningfulness of anti-cN-1A testing.

## Abbreviations

cN-1A: Cytosolic 5′-nucleotidase 1A
ELISA: Enzyme-linked immunosorbent assay
Ig: Immunoglobulin
IIM: Idiopathic inflammatory myopathy
sIBM: Sporadic inclusion body myositis
